# Identification of P-Rex1 as an anti-inflammatory and anti-fibrogenic target for pulmonary fibrosis

**DOI:** 10.1038/srep25785

**Published:** 2016-05-13

**Authors:** Qing Liang, Ni Cheng, Gufang Zhang, Yurong Liang, Feng Qian, Dianqing Wu, Richard D. Ye

**Affiliations:** 1 School of Pharmacy, Shanghai Jiao Tong University, Shanghai 200240, China; 2 Department of Pharmacology, University of Illinois College of Medicine, Chicago 60612, USA; 3 Department of Pharmacology, Yale School of Medicine, New Heaven, CT 06510, USA; 4 Institute of Chinese Medical Sciences and the State Key Laboratory of Quality Research in Chinese Medicine, University of Macau, Macau SAR, China

## Abstract

Pulmonary fibrosis (PF) leads to progressive and often irreversible loss of lung functions, posing a health threat with no effective cure. We examined P-Rex1, a PI3K- and G protein βγ-regulated guanine nucleotide exchange factor (GEF) of the Rac small GTPase, for its potential involvement in PF. In a bleomycin-induced PF model, mice deficient in *p-rex1* had well-preserved alveolar structure and survived significantly better than their wild type (WT) littermates. The *p-rex1*^−/−^ mice expressed significantly less proinflammatory cytokines and chemokines and had reduced leukocyte infiltration in the lung tissue than their WT littermates. P-Rex1 was detected in lung fibroblasts of WT mice, and its genetic deletion attenuated TGFβ-1-stimulated lung fibroblast migration, Rac1 activation and p38 MAPK phosphorylation. The *p-rex1*^−/−^ mice showed significantly reduced pathological changes including the expression of α-smooth muscle actin, fibronectin and TGFβ-1 compared with their WT controls. Expression of a GEF-deficient P-Rex1 mutant effectively blocked Smads-dependent transcriptional activation, suggesting that P-Rex1 is a downstream mediator of TGFβ-1 signaling. These findings identify P-Rex1 as a novel player of PF, suggesting that targeting P-Rex1 may simultaneously block the inflammatory and fibrogenic processes of PF.

Pulmonary fibrosis (PF) is a chronic disorder that leads to progressive loss of lung functions with a high mortality rate. PF may be a consequence of radiation, chemotherapy and occupational inhalation of dust particle, or without a clearly identifiable cause (idiopathic pulmonary fibrosis, IPF)^1^. All forms of PF are characterized by increased synthesis and deposit of extracellular matrix (ECM) proteins including collagen and fibronectin, accompanied by elevated production of transforming growth factor β 1 (TGF-β1) and increased proliferation of interstitial fibroblasts^2^. Despite detailed characterization of the pathological changes in PF, however, an effective cure for PF remains elusive. Hence, identification of novel targets for PF is crucial to the development of therapies for PF. Several mouse models have been developed for research on PF^3^. Among these models, bleomycin-induced fibrosis in rodents has been widely used in research laboratories. Bleomycin is an antineoplastic drug that binds DNA^4^. Intratracheal administration of bleomycin results in cell death within 24 hours. On days 2 and 3 following bleomycin administration, inflammation of the lung (alveolitis) occurs and is gradually developed into interstitial inflammation on days 4–12. The fibrogenic response, characterized with fibroblast proliferation and increased production of ECM proteins, begins on day 4 after bleomycin administration and culminates on Day 21^5^. Using this model, several factors that affect the inflammatory and fibrogenic phases of PF have been identified. For instance, the use of the bleomycin-induced PF model has accelerated our understanding of the *in vivo* functions of TGF-β1 in fibrosis^6^.

One of the limiting factors in developing an effective therapy for PF is the lack of understanding of the transition from the inflammatory phase to the fibrogenic phase. Multiple factors contribute to the development of IPF, but many of them, including TGF-β and Smad 3, are not suitable targets for long-term drug intervention due to their pleotropic functions^6^. We investigated whether P-Rex1, a PI3K- and Gβγ-regulated guanine nucleotide exchange factor (GEF) known for its roles in the activation of Rac^7^,^8^, is involved in the development of PF in bleomycin-treated mice. P-Rex1 is a Dbl-family GEF initially identified in neutrophils and neurons^7^. Its functions in the regulation of the inflammatory response (reviewed in^8^) led us to investigate a potential role for P-Rex1 in the development of PF. Our work show that P-Rex1 is not only involved in the inflammatory response resulting from bleomycin-induced lung damage, but also contributes to the fibrogenic response of PF by acting as a downstream effector for TGF-β1 signaling. Mouse survival assay and histological analysis indicate that genetic deletion of *p-rex1* offers protection against bleomycin-induced PF, as evidenced by reduced TGF-β1 production, abrogated fibroblast migration and reduced mortality. These findings offer the possibility of targeting P-Rex1 for PF therapy based on its dual functions in the inflammatory and fibrogenic processes.

## Results

### P-Rex1 deficiency attenuates bleomycin-induced pulmonary fibrosis and reduces mortality in afflicted mice

**Figure 1:**
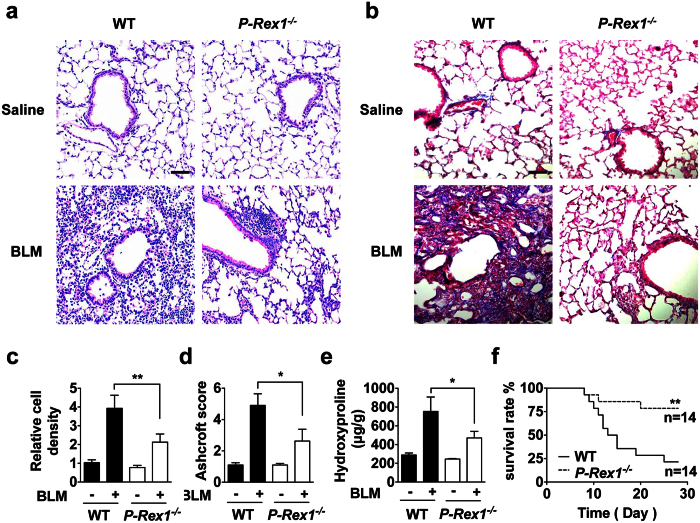
P-Rex1 deficiency attenuates bleomycin-induced pulmonary fibrosis and reduces mortality in mice. Eight-week-old male WT and *p-rex1*^−/−^ mice (6–8 each group) were given bleomycin sulfate in saline (1.2 U/kg body weight) or saline alone, by intratracheal injection in a 50 μl total volume. Lungs were removed from mice for fibrosis analysis 21 days post challenge. Shown in this figure are representative images of H&E staining (**a**) and Masson’s trichrome staining (**b**) of lung sections obtained from WT and *p-rex1*^−/−^ mice with bleomycin (BLM) or saline administration. Scale bars = 100 μm. (**c**) Total cell counts in the lung sections stained with H&E. (**d**) Ashcroft score of the lung sections from WT and *p-rex1*^−/−^ mice. (**e**) Hydroxyproline content of lungs was determined after saline or BLM administration to WT and *p-rex1*^−/−^ mice. (**f**) Survival rate of WT and *p-rex1*^−/−^ mice after bleomycin challenge (2.0 U/kg body weight) determined in a 28 d period. Data shown in (**c–e**) are means ± SEM using at least 5 mice. (*p < 0.05 and **p < 0.01).

Mice were given bleomycin sulfate to induce the development of pulmonary fibrosis^3^. Twenty-one days after intratracheal injection of a single dose of bleomycin, histological analysis of the WT mice lungs showed destruction of alveolar architecture, massive collagen deposit, and a marked increase in the number of fibroblasts. In comparison, lung sections from the *p-rex1*^−/−^ mice showed well-preserved alveolar structure, fewer fibroblasts and less collagen deposition around capillary vessels (Fig. 1a–c). Ashcroft scores taken on day 21 were significantly different between the *p-rex1*^−/−^ mice and their WT littermates (Fig. 1d). Moreover, a significant decrease in hydroxyproline level, representing total lung collagen content, was detected in bleomycin treated *p-rex1*^−/−^ mice compared to WT mice (Fig. 1e). More importantly, a much improved survival rate was seen in *p-rex1*^−/−^ mice than WT mice 28 days after the administration of bleomycin (Fig. 1f).

**Figure 2:**
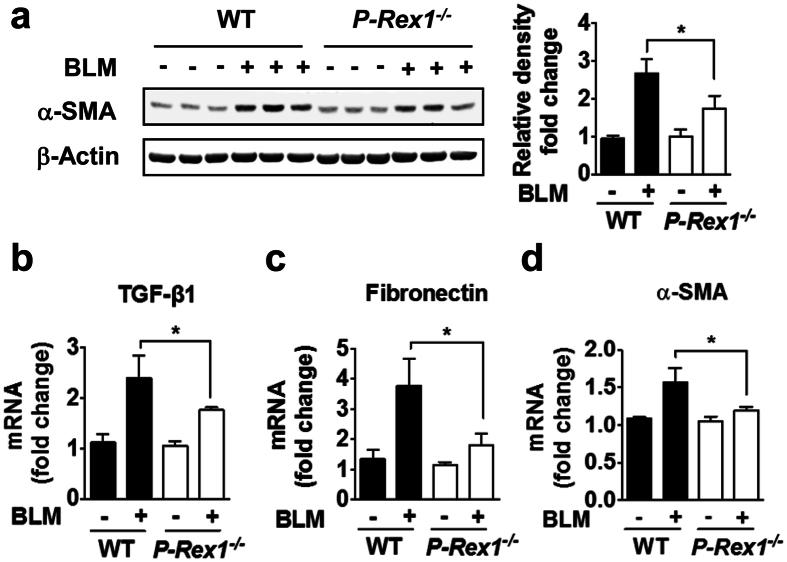
Down-regulation of α-SMA and fibronectin in bleomycin treated *p-rex1*^−/−^ mice compared with WT mice. Lungs were removed from mice on day 21 after bleomycin administration and were homogenized. (**a**) α-SMA protein expression in lung tissues from WT and *p-rex1*^−/−^ mice with bleomycin (BLM) or saline administration, shown in representative Western blots (left) and bar chart (right). The transcripts for mouse TGF-β1 (**b**), fibronectin (**c**) and α-SMA (**d**) were determined in the lung tissue from WT and *p-rex1*^−/−^ mice with bleomycin or saline administration. Data shown are means ± SEM (*p < 0.05) with n = 6–8 per group.

Western blot analysis of the total lung homogenates from WT mice showed that bleomycin administration increased the expression of α-smooth muscle actin (SMA), a myofibroblast marker indicative of increased collagen gene expression^9^,^10^. Comparing to the WT controls, the *p-rex1*^−/−^ mice showed a marked reduction in α-SMA expression (Fig. 2a). Likewise, the expression of several markers of PF including TGF-β1 and fibronectin was also blunted in *p-rex1* deficient mice (Fig. 2b–d). These results suggest that P-Rex1 deficiency ameliorates bleomycin-induced PF in mice.

### The bleomycin-induced lung inflammatory response is reduced in *p-rex1* ^−/−^ mice

**Figure 3:**
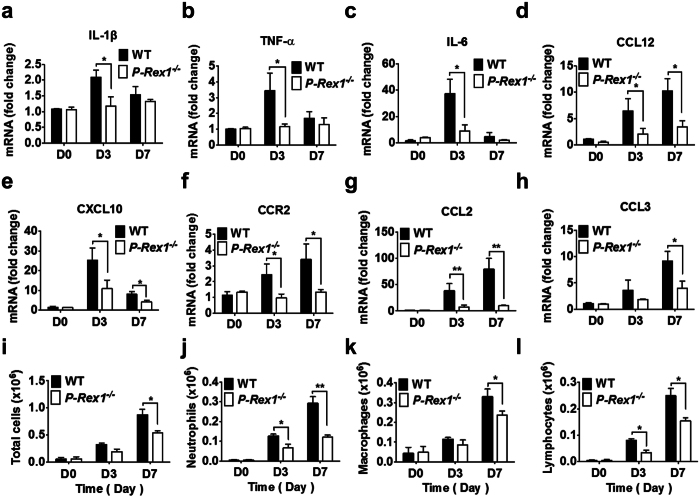
Lung tissue expression of cytokines and chemokines and leukocyte infiltration in bleomycin-treated WT and *p-rex1*^−/−^ mice. On days 0, 3 and 7 after bleomycin challenge, mice were sacrificed and lung tissue homogenates were examined by Q-PCR. Bronchoalveolar lavage fluid (BALF) was collected for total and differential cell counts. (**a–h**) transcripts for IL-1β, TNF-α, IL-6, CCL12, CXCL10, CCR2, CCL2 and CCL3 in WT and *p-rex1*^−/−^ mouse lung tissue homogenates. Total cell counts (**i**), cell counts for neutrophils (**j**), macrophages (**k**) and lymphocytes (**l**) were determined in the BALF of WT and *p-rex1*^−/−^ mice. Data shown are means ± SEM. *p < 0.05 and **p < 0.01, n = 6–8 per group.

Bleomycin causes damage to the alveolar epithelial cells, leading to an interstitial inflammatory response within the first week after its administration^11^,^12^. We compared the expression of selected inflammatory cytokines and chemokines in lung tissue homogenates as well as leukocyte accumulation in the bronchoalveolar lavage fluid (BALF) of WT and *p-rex1*^−/−^ mice, on days 0, 3 and 7 after bleomycin challenge. At the mRNA level, the proinflammatory cytokines IL-1β, TNF-α and IL-6 were significantly elevated on day 3 after bleomycin administration in WT mice; in comparison, the up-regulation of these cytokines was less pronounced in *p-rex1*^−/−^ mice (Fig. 3a–c). In the course of PF, CCL12, CXCL10 and CCR2 are major chemokines for fibrocytes recruitment from bone marrow to the lung tissue for differentiation into fibroblasts^13^,^14^. The expression of these chemokines, along with CCL2 and CCL3, were significantly increased on days 3 and 7 after bleomycin administration in WT mice. In contrast, the induction of these chemokines was significantly attenuated in the *p-rex1*^−/−^ mice (Fig. 3d–h). The bleomycin-induced inflammatory cell infiltration into the lung tissue was determined by cell counting in bronchoalveolar lavage fluid (BALF), collected from WT and *p-rex1*^−/−^ mice treated with bleomycin or saline. Total cell counts were changed on days 3 and 7, with statistical significance observed on day 7 between the samples (Fig. 3i). The cell counts for neutrophils (Fig. 3j), macrophages (Fig. 3k) and lymphocytes (Fig. 3l) in the BALF were also attenuated in the *p-rex1*^−/−^ mice compared to the WT mice. Altogether, these findings demonstrate that deletion of *p-rex1* reduced the inflammatory response to bleomycin.

### P-Rex1 deficiency attenuates pulmonary fibrosis through decreased Rac1 activation in lung fibroblasts

**Figure 4:**
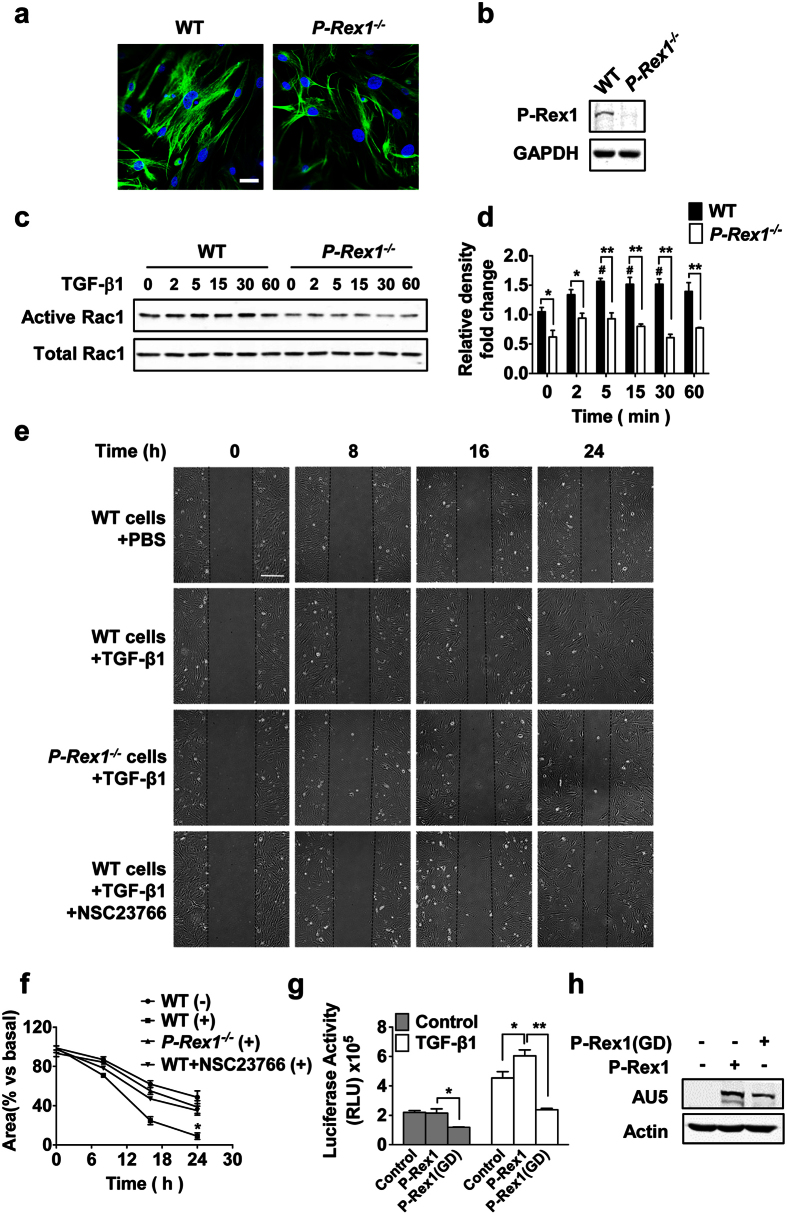
P-Rex1 is involved in TGF-β signaling in lung fibroblasts. (**a**) Cultured fibroblasts from WT and *p-rex1*^−/−^ adult mice were stained for vimentin (green fluorescence). Representative images are shown; scale bars = 25 μm. (**b**) A representative Western blot showing P-Rex1 in WT but not *p-rex1*^−/−^ fibroblasts. (**c**) Activation of Rac1 after mouse TGF-β1 (10 ng/ml) stimulation for the indicated time (min) in WT and *p-rex1*^−/−^ lung fibroblasts. (**d**) Quantification of data in (**c**) and 3 similar Western blots (not shown). The level of activated Rac1 in each sample was normalized against the integrated density of total Rac1 present in each sample. *p < 0.05 and **p < 0.01 comparing *p-rex1*^−/−^ fibroblasts with WT fibroblasts in the same stimulation groups; ^#^*p* < 0.05 comparing TGF-β1-stimulated fibroblasts with unstimulated fibroblasts of the same genotype. (**e**) Wound closure with serum-starved WT and *p-rex1*^−/−^ fibroblasts, treated with or without NSC23766 (100 μM) for 1 h and then stimulated with or without mouse TGF-β1 (10 ng/ml) for a total of 24 h. Microphotographs were taken at 0, 8, 16 and 24 h after scratching. Scale bars = 25 μm. (**f**) Analysis of wound closure area in WT and *p-rex1*^−/−^ fibroblasts. *p < 0.05 compared with *p-rex1*^−/−^ (+). (**g**) Requirement of P-Rex1-dependent guanine nucleotide exchange for TGF-β1-induced SBE4-Luc expression. HEK293T cells were co-transfected with the SBE4-Luc together with an AU5-tagged P-Rex1 expression construct or a similarly tagged GEF-dead (GD, E56A/N238A) mutant construct of P-Rex1 or an empty vector (control). The cells were stimulated with human TGF-β1 (10 ng/ml) for 16 h, and luciferase activity was examined. (**h**) A representative Western blot showing the expression of the AU5-P-Rex1 and AU5-P-Rex1 (GD) in transfected HEK293T cells. Quantitative data shown above are means ± SEM from 3 independent experiments. (*p < 0.05 and **p < 0.01).

Fibroblasts play a pivotal role in maintaining normal lung functions as well as in tissue repair. In the presence of TGF-β1, fibroblasts differentiate into myofibroblasts, which secrete extracellular matrix proteins that results in the development of pulmonary fibrosis. Having established a role for P-Rex1 in the early inflammatory phase of bleomycin-induced pulmonary fibrosis, our next experiment was focused on the effect of P-Rex1 deficiency in lung fibroblasts. Lung fibroblasts were isolated from WT and *p-rex1*^−/−^ mice. These cells display the characteristic spindle shape morphology and positive staining for vimentin (Fig. 4a) and were used in the experiments. P-Rex1 expression was detected in fibroblasts from the lungs of WT mice, but not those from *p-rex1*^−/−^ mice (Fig. 4b). It should be mentioned that although P-Rex1 was originally identified in neutrophils and neurons^7^,^15^, its expression is much broader as shown in studies using endothelial cells and several types of cancer cells^16^,^17^.

P-Rex1 primarily functions as a GEF for Rac1 activation. Therefore, endogenous Rac1 activity was first examined in fibroblast stimulated with mouse TGF-β1. In WT fibroblasts, a persistent and statistically significant increase in Rac1 activation was observed after TGF-β stimulation. The level of Rac1 activation was much reduced in *p-rex1*^−/−^ fibroblasts (Fig. 4c,d). These findings provide the first evidence for a role of P-Rex1 in TGF-β1-stimulated Rac1 activation in lung fibroblasts. TGF-β1 is well known for its potent chemotactic effect^18^. To assess the cellular functions of P-Rex1, fibroblast migration was analyzed using an *in vitro* wound-healing assay^19^. The TGF-β1-dependent fibroblast migration was visibly slower in *p-rex1*^−/−^ fibroblasts than in WT fibroblasts. Treatment of WT fibroblasts with the Rac1 inhibitor NSC23766 produced results similar to that of *p-rex1* deficiency (Fig. 4e,f), suggesting that the observed difference in cell migration was due to P-Rex1-dependent Rac1 activation. To further validate the involvement of P-Rex1 in TGF-β1-induced Rac1 activation, luciferase activity assay was performed using a TGF-β1 pathway reporter gene, the Smad binding element 4 (SBE4)-Luc, which is downstream of Smad3 and Smad4^20^. When HEK293T cells were co-transfected with both SBE4-Luc and an expression vector for P-Rex1, TGF-β1 treatment induced a significant increase in luciferase activity above the base level. Expression of a P-Rex1 mutant devoid of GEF activity (GEF-dead, GD)^21^ together with SBE4-Luc not only abrogated the TGF-β1 induced luciferase activity, but also significantly reduced TGF-β1 responsiveness at basal condition, suggesting that P-Rex1 (GD) serves as a dominant negative mutant that blocked the TGF-β1-induced Rac1 activation through P-Rex1 (Fig. 4g,h). These findings establish a cellular function of P-Rex1 in TGF-β1 signaling that leads to Rac-dependent fibroblast migration and Smads-dependent transcriptional activation.

### p38 MAPK is downstream of Rac1 and regulates TGF-β1 secretion in mouse lung fibroblasts

**Figure 5:**
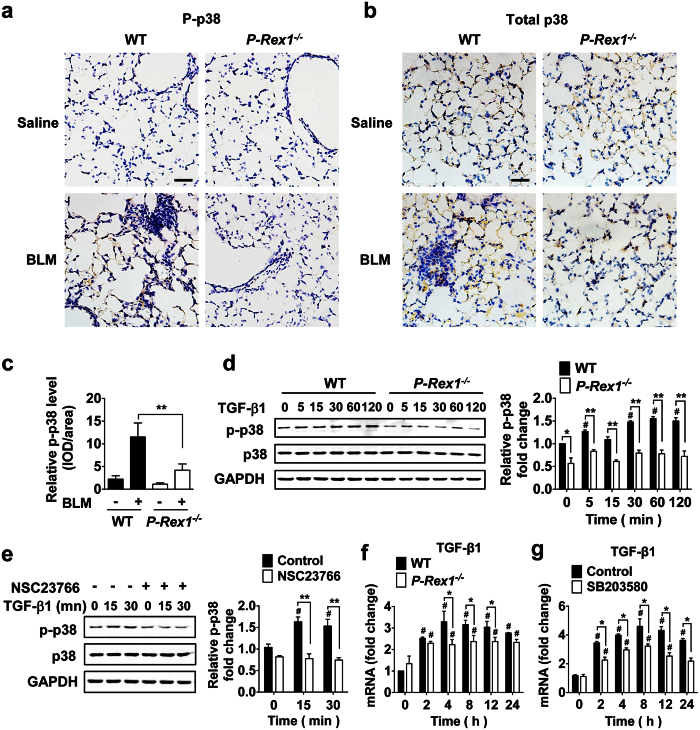
P-Rex1 is required for TGF-β-induced phosphorylation of p38 MAPK. (**a,b**) Representative images showing immunohistochemistry staining for phosphorylated p38 MAPK (p-p38, (**a**)) and total p38 MAPK (**b**) in lung sections from WT and *p-rex1*^−/−^ mice receiving bleomycin (BLM, same as in Fig. 1) or saline alone. Scale bars = 100 μm. (**c**) Quantification of p-p38 in the above lung sections. The p-p38 level in each sample was calculated as the ratio of integrated optical density (IOD) to area, and normalized against total p38 MAPK present in the same sample. Data shown are means ± SEM based on at least 5 mice. (**d**) Phosphorylation of p38 MAPK after TGF-β1 (10 ng/ml) stimulation for the indicated time (min) in WT and *p-rex1*^−/−^ lung fibroblasts. The relative phosphorylation level of p38 MAPK in each sample was normalized against the integrated density of total p38 MAPK present in each sample. (**e**) WT mouse lung fibroblasts were pre-incubated with NSC23766 (100 μM) for 1 h before TGF-β1 (10 ng/ml) stimulation for the indicated time (min). Cell lysates were subjected to SDS-PAGE and Western blotting for p-p38 and total p38 MAPK. A representative set of images from 3 similar experiments is shown. Data quantification is shown in the right panel. (**f**) Mouse TGF-β1 mRNA expression in WT and *p-rex1*^−/−^ lung fibroblasts after TGF-β1 (10 ng/ml) stimulation for the indicated time periods. (**g**) Same as in (**f**) but with WT fibroblasts exposed to SB203580 (10 μM) or vehicle for 1 h before stimulation with mouse TGF-β1. Data shown in bar charts (**d–g**) are means ± SEM from at least 3 independent experiments. *p < 0.05 and **p < 0.01 comparing *p-rex1*^−/−^ fibroblasts (or drug treated sample) with WT fibroblasts (or untreated sample) in the same time group; ^#^*p* < 0.05 comparing TGF-β1-stimulated fibroblasts with unstimulated fibroblasts of the same genotype.

The p38 MAPK is a component of the TGF-β1-activated signaling pathways^6^,^22^. Published reports show that p38 MAPK plays a role in bleomycin-induced fibrosis^23^,^24^. We performed an immunohistochemical analysis of lung sections and found that bleomycin induced an increase in the relative level of phosphorylated p38 MAPK (p-p38/total p38, Fig. 5a,b). Moreover, there was a significant difference in the relative level of p-p38 between the WT lung sections and *p-rex1*^−/−^ lung sections (Fig. 5c). Results obtained with cultured lung fibroblasts from WT and *p-rex1*^−/−^ mice confirmed that the TGF-β1-induced p38 phosphorylation was abolished in the absence of P-Rex1 (Fig. 5d). Likewise, treatment of lung fibroblasts with the Rac inhibitor NSC 23766 abolished the TGF-β1-nduced phosphorylation of p38 MAPK (Fig. 5e). Given the established role for TGF-β1 in lung fibrosis^25^, we sought to determine whether P-Rex1/Rac contributes to the TGF-β1 autocrine loop that promotes trans-differentiation of fibroblast to myofibroblast^26^,^27^. In WT lung fibroblasts, the expression level of TGF-β1 was markedly induced with the treatment of TGF-β1, but the induction was attenuated in *p-rex1*^−/−^ lung fibroblasts (Fig. 5f). Treatment of lung fibroblasts with the p38 inhibitor SB 203580 prior to TGF-β1 stimulation produced a similar effect in attenuating the induced expression of TGF-β1 (Fig. 5g). Collectively, these findings suggest that P-Rex1-dependent activation of Rac is required for the TGF-β1-induced phosphorylation of p38 MAPK that partially contributes to autocrine regulation of TGF-β1 expression in fibroblasts.

## Discussion

Using the bleomycin-induced PF model, we identified P-Rex1 as a novel player in the pathogenesis of PF. Mice lacking *p-rex1* survived much better than their WT littermates when exposed to bleomycin, suggesting that these mice are refractory to bleomycin-induced inflammatory and fibrogenic responses. The two phases of PF development have distinct pathological features but are closely connected with an overlapping period. The inflammatory response to bleomycin-induced injury becomes evident 3 days after bleomycin administration, and the fibrogenic response follows a few days later. In each of these phases, the *p-rex1* deficient mice showed significantly reduced pathological changes than their WT littermates. Three days after bleomycin administration, the expression of proinflammatory cytokines including IL-1β, TNF-α and IL-6 reached peak level in WT mice. Among the chemokines examined, the expression of CXCL10 also reached peak level on day 3 after bleomycin administration. In comparison, significantly lower levels of the proinflammatory cytokines were seen in the lungs of the *p-rex1*-deficient mice. Of note, the expression level of the two major inflammatory cytokines, IL-1β and TNF-α, was as low as in unchallenged mice, demonstrating that P-Rex1 is critical to the induction of these proinflammatory cytokines. The expression level of CCR2, CCL2, CCL3 and CCL12 continued to rise after day 3 with higher levels of expression detected on day 7 after bleomycin administration. Again, the *p-rex1*^−/−^ mice showed much lower induction of these chemokines than their WT littermates. These results, combined with the reduced infiltration of neutrophils, macrophages and lymphocytes in the lung tissue of the *p-rex1*^−/−^ mice, demonstrate a critical role for P-Rex1 in lung inflammation following bleomycin-induced injury. Our observations are consistent with an established role for P-Rex1 in inflammatory cells, including neutrophils^28^,^29^, platelets^30^ and microvascular endothelial cells^31^.

Our experimental data unveil a new function of P-Rex1 in the fibrogenic process. In addition to a reduced expression of α-SMA, a marker of myofibroblasts, the *p-rex1* deficient mice showed a nearly intact alveolar structure, reduced collagen contents in the lungs, and significantly lower levels of fibronectin and TGF-β1. Moreover, the TGF-β1-stimulated Rac activation was significantly decreased in *p-rex1*^−/−^ fibroblasts, and the TGF-β1 signaling pathways leading to p38 MAPK phosphorylation in the WT fibroblasts was abrogated in the *p-rex1* deficient mice. Of note, TGF-β1 induced a rapid activation of p38 MAPK within 5 min, and a sustained p38 MAPK phosphorylation that started after 15 min. Sustained phosphorylation of p38 MAPK was also observed in the mouse lung sections 21 days after bleomycin administration. In other tissues, inhibition of p38 MAPK could prevent the TGF-β1-induced myofibroblast trans-differentiation^32^, thus providing strong evidence for the involvement of p38 MAPK in the fibrogenic process. Altogether, these findings strongly support a role for P-Rex1 and the resulting activation of the small GTPase Rac in the activation of p38 MAPK following TGF-β1 stimulation.

P-Rex1 was originally identified as a PI3K- and G protein βγ-regulated guanine nucleotide exchange factor for the Rac small GTPases. It is primarily involved in GPCR signaling, but also plays a role in cancer cell metastasis^16^,^33^ and TNF-α induced increase in lung endothelial permeability^31^. Our results show for the first time that P-Rex1 is involved in TGF-β1 signaling, suggesting that P-Rex1 may directly or indirectly contribute to myofibroblast differentiation and expression of specific markers for PF. The TGF-β1-stimulated PI3K activity may be sufficient for P-Rex1 activation, as there has been no evidence that TGF-β1 signaling involves any Gβγ activation. A P-Rex1/Rac activation mechanism in lung fibroblasts is supported by the observations that inhibition of the Rac small GTPase with NSC 23766 and expression of a GEF-dead P-Rex1 mutant produced similar outcomes in the *in vitro* wound-healing assay. Based on these results as well as the current understanding of the TGF-β1 signaling pathways^6^, we postulate that P-Rex1 is a proximal regulator of TGF-β1 signaling. Several observations made in the *p-rex1* deficient fibroblasts support a role for P-Rex1 in TGF-β1-induced signaling including Rac activation: (1) a reduction in TGF-β1-induced p38 MAPK phosphorylation in these cells suggest that p38 MAPK activation downstream of TGF-β1 requires P-Rex1-dependent Rac activation; (2) a reduction in TGF-β1 induced wound closure implies that the P-Rex1/Rac activation mechanism contributes to fibroblast migration, which is critical to scar formation *in vivo*; (3) a reduction in α-SMA expression suggests an involvement of P-Rex1 in the TGF-β1 signaling pathways leading to SBE activation; (4) our finding that a GEF-dead mutant of P-Rex1 could suppress TGF-β1-induced expression of SBE-Luc in a well-defined transfection system also suggested an indirect function of P-Rex1 in the activation of the Smads-SBE pathway. These findings have opened opportunities for further investigation into the exact role for P-Rex1 in TGF-β1 signaling.

Pulmonary fibrosis remains a major health concern that requires continued research effort for the development of effective therapies. The identification of P-Rex1 as a signaling molecule involved in both the inflammatory and fibrogenic pathways of PF may shed light on the mechanisms by which cells in the lung respond to acute injury. Moreover, P-Rex1 may be a potential drug target because of its dual functions in the inflammatory and fibrogenic processes. Further investigation into the exact role for P-Rex1 in PF may facilitate the design of novel approaches for better clinical treatment.

## Methods

### Animals

The generation and characterization of *p-rex1* knockout mice was described previously^28^. *P-rex1*^−/−^ mice and their *p-rex1*^+/+^ (WT) littermates were housed under specific pathogen free conditions. All procedures involving the use of the mice were carried out in accordance with the *Statute on the Administration of Laboratory Animals* by the National Science & Technology Council of China, using protocols approved by the Institutional Animal Care and Use Committee of Shanghai Jiao Tong University. For bleomycin administration, 8-week-old male WT and *p-rex1*^−/−^ mice (6–8 each group) were anesthetized and given bleomycin sulfate (BIOTANG Inc., Lexington, MA, USA) by intratracheal injection at 1.2 U/kg body weight for fibrosis model, or 2 U/kg body weight for lethality. These mice were allowed to recover after bleomycin administration. WT and *p-rex1*^−/−^ mice were euthanized for fibrogenic analysis on day 21 and inflammatory analysis on days 0, 3 and 7 after a single dose of bleomycin treatment.

### Histopathology and Hydroxyproline analysis of PF

The left lungs were removed from WT and *p-rex1*^−/−^ mice on day 21 after bleomycin administration and immediately fixed in 10% neutral buffered formalin for at least 24 h. The fixed lungs were sectioned, embedded in paraffin and cut into 5 μm sections for hematoxylin and eosin (H&E) or Masson staining according to the manufacturer’s instructions (Nanjing Jiancheng Bioengineering Institute, Nanjing, China). The hydroxyproline content was measured based on alkaline hydrolysis of the lung tissues and performed as per manufacturer’s instructions (Nanjing Jiancheng Bioengineering Institute).

### Immunohistochemistry

Paraffin sections of WT and *p-rex1*^−/−^ mouse lung tissue were subjected to deparaffinization, rehydration and antigen retrieval steps. The sections were incubated in 3% hydrogen peroxide for 10 min to block endogenous peroxidase activity. Total p38 MAPK and phosphorylated p38 MAPK (p-p38) were detected with antibodies against these forms of p38 MAPK, respectively, by incubating the sections with a 1:100 dilution of each of the antibodies overnight at 4 °C. The sections were then incubated for 1 h at room temperature with HRP-conjugated anti-rabbit antibody, followed by counterstaining with hematoxylin. The level of stained p38 MAPK was semi-quantitatively measured as the integrated optical density (IOD), using the Image-pro plus Software (Media Cybernetics, Silver Spring, MD, USA). The relative level of p38 MAPK phosphorylation in the tissue sections is defined as (IOD of p-p38/area)/(IOD of total p38/area).

### Isolation and culture of primary murine lung fibroblasts

Fibroblasts from adult mice were isolated as previously described^34^. Briefly, 6-to 8-week old WT or *p-rex1*^−/−^ mice were euthanized and the whole lungs were obtained, cut into small pieces, and then subjected to collagenase (type IV, Sigma) digestion for 30 min in DMEM with 100 IU/ml penicillin and 100 μg/ml streptomycin. After centrifugation, the pellets containing tissue fragments and cells were washed twice and finally placed in 100-mm dishes for culture in DMEM containing 10% FBS with 100 IU/ml penicillin and 100 μg/ml streptomycin. After 7 days, tissue fragments were discarded and cells were harvested for experiments. The cultured fibroblasts were identified by their expression of vimentin.

### Rac activation assay

Rac activation assay was performed based on the affinity of Rac-GTP for the p21-binding domain of PAK1 as previously described^35^,^36^. Briefly, a GST tagged PBD of PAK1 fusion protein was immobilized on Glutathione Sepharose 4B beads (GE Healthcare, Piscataway, NJ, USA). Fibroblast lysate was incubated with 20 μg of GST tagged PBD protein overnight at 4 °C. Beads were washed five times with wash buffer and resuspended in SDS sample loading buffer for analysis by Western blotting, using an anti-Rac1 antibody (Thermo Scientific, Waltham, MA, USA) for detection.

### Wound-healing assay

WT and *p-rex1*^−/−^ fibroblasts isolated from adult mice lungs were placed in six-well plates and grown to confluence. Serum-starved cells were scratched using a standard 20-μl pipette tip and then stimulated with or without mouse TGF-β1 (10 ng/ml) for 24 h. Fibroblasts migration was evaluated by measuring reduced wound area *vs*. total area in a 24 h observation period. Microphotographs were taken at 0, 8, 16 and 24 h after scratch. To examine the effect of Rac inhibition, the cells were pre-incubated with NSC23766 (100 μM) for 1 h before scratch.

### Luciferase activity assay

HEK293T cells were co-transfected with SBE4-Luc with or without AU5-tagged human P-Rex1 or a similarly tagged GEF-deficient (GD, E56A/N238A) construct of human P-Rex1 plasmid^21^. About 24 h post-transfection, cells were treated with or without (Control) human TGF-β1 (10 ng/ml) for 16 h and then harvested and lysed in lysis buffer. Luciferase activity was analyzed using a Dual-Luciferase Reporter Assay Kit according to the manufacturer’s instructions (Promega, Fitchburg, WI).

### Bronchoalveolar lavage fluid (BALF)

WT and *p-rex1*^−/−^ mice were anesthetized and lungs were lavaged twice with 1 ml cold PBS on days 0, 3 and 7 after bleomycin administration. Red blood cells were lysed, and the remained cells were washed twice with PBS and collected for total cell counts. Differential cell counts were determined with Wright-Giemsa staining (Nanjing Jiancheng Bioengineering Institute, Nanjing, China) on cytospin slides according to the manufacturer’s instructions (200 cells per animal were examined).

### Statistical analysis

Data were shown as means ± SEM from at least 3 independent experiments. The Differences between groups were subjected to one-way ANOVA between indicated groups. Survival analysis was performed using log-rank (Mantel-Cox) test. Values of *p* < 0.05 were considered statistically significant.

## Additional Information

**How to cite this article**: Liang, Q. *et al*. Identification of P-Rex1 as an anti-inflammatory and anti-fibrogenic target for pulmonary fibrosis. *Sci. Rep.* **6**, 25785; doi: 10.1038/srep25785 (2016).
